# Dicer is cleaved by the Leader protease encoded by foot-and-mouth disease virus to promote infection in mammalian cells

**DOI:** 10.1126/sciadv.adt3751

**Published:** 2025-07-04

**Authors:** Miguel Rodríguez-Pulido, Miguel Ángel Sanz, Lucía Camacho, Ricardo Ramos, Margarita Sáiz

**Affiliations:** ^1^ Centro de Biología Molecular Severo Ochoa, CSIC-UAM, Madrid, Spain.; ^2^ Genomic Facility, Fundación Parque Científico de Madrid, Madrid, Spain.

## Abstract

The endoribonuclease Dicer is a central component of the posttranscriptional gene silencing mechanism based on RNA interference (RNAi) in eukaryotes. The antiviral role of RNAi in mammalian cells remains controversial while a number of viral suppressors of RNAi (VSR) able to inhibit Dicer activity and promote infection have been identified. Here, we explored the integrity and functional role of Dicer during FMDV infection. These studies showed that the FMDV-encoded Leader protease (Lpro) specifically cleaves Dicer at a conserved DExD/H helicase motif releasing the complete N-terminal helicase domain. Dicer cleavage by Lpro suppressed small hairpin RNA (shRNA)–induced RNAi in swine cells. Silencing of Dicer conferred increased susceptibility to an Lpro-deficient FMDV, revealing a Dicer-dependent antiviral effect which can be effectively counteracted by Lpro. This mutant generated a remarkably different profile of viral small RNAs (vsRNAs) in infected cells compared with the wild-type virus. Overall, we identified a viral mechanism of dampening or modulating antiviral defenses based on Dicer proteolytic degradation.

## Teaser

Dicer is targeted for cleavage by the FMDV-encoded Leader protease to circumvent its antiviral role in mammalian cells.

## INTRODUCTION

RNA interference (RNAi) is a highly conserved antiviral immune mechanism in eukaryotes that plays an essential role in plants and invertebrates (*1*). In RNAi, double-stranded RNA (dsRNA) produced during RNA virus infection is cleaved by the cytoplasmic host protein Dicer into 21- to 23-nt-long small interfering RNAs (siRNAs), which are incorporated into the RNA-induced silencing complex (RISC) and guide the binding to complementary viral RNA to induce its degradation (*2*, *3*). Dicer is also involved in microRNA (miRNA) biogenesis by cleavage of precursor miRNAs (pre-miRNAs) and regulation of cellular gene expression through the miRNA-mediated gene silencing pathway (*4*, *5*). The functional relevance of the RNAi pathway in antiviral immunity operating in mammalian differentiated cells remains a subject of intense debate. The antiviral response in mammalian cells is primarily orchestrated by the secretion of type-I and type-III interferons (IFNs). The dsRNA derived from viral infection is detected by RIG-I–like receptors (RLRs), which include RIG-I (retinoic acid–inducible gene I), MDA5 (melanoma differentiation factor 5), and LGP2 (laboratory of genetics and physiology 2) (*6*). A signaling cascade is then activated leading to IFNs expression. IFNs then trigger in an autocrine and paracrine manner the transcription of hundreds of IFN-stimulated genes (ISGs) encoding proteins with antiviral function focused on degradation of viral nucleic acid or inhibition of viral gene expression (*7*). RNA viruses often encode proteins antagonizing host immunity. Foot-and-mouth disease virus (FMDV) is a remarkable example of that, exerting a number of mechanisms at different steps and signaling routes that enable the rapid propagation of the pathogen (*8*–*10*). FMDV belongs to the family Picornaviridae including small nonenveloped positive-stranded RNA viruses and is the causal agent of a highly infectious disease affecting farm and wild animals worldwide with a significant economic impact (*11*). Among the immune evasion strategies evolved by FMDV, the role of the papain-like cysteine Leader protease (Lpro) in counteracting the host immune response has proven crucial for pathogenesis (*12*). Lpro is located at the N terminus of the polyprotein and releases itself by cleavage at its own C terminus. Lpro is expressed as two forms, Lab and Lb, by translation initiation at two AUG codons (AUG1/2) separated 84 nt on the viral genome, being Lb the most abundant in FMDV-infected cells (*13*). The proteolytic activity of Lpro is implicated in suppressing IFN-α/β induction, and a number of proteins have been identified as Lpro targets (*14*). Early in infection, Lpro cleaves the translation initiation factors eIF4GI and eIF4GII, inducing the shutdown of cap-dependent translation (*15*). Lpro is known to induce the degradation of nuclear factor κB (NF-κB) subunit p65/RelA and reduce interferon regulatory factor 3 (IRF3)/7 expression (*16*, *17*). Direct cleavage of viral sensors LGP2 and MDA5 by Lpro has been documented and associated to impairment of the antiviral response against FMDV infection (*18*, *19*). Also, MAVS and TBK1, relevant proteins in the IFN pathway, have been identified as Lpro targets (*20*). The cleavage/degradation of RLR signaling proteins, but not the deISGylase/DUB activity reported for Lpro has been correlated with suppressing IFN-α/β gene transcription (*20*). The counteracting effect of Lpro on the DNA sensing cyclic Guanosine monophosphate-Adenosine monophosphate Synthase/Stimulator of IFN Genes (cGAS/STING) pathway, also involved in antiviral response against RNA viruses, has been recently documented (*21*).

The IFN system is known to actively inhibit dsRNA-mediated RNAi and a truncated Dicer isoform with enhanced antiviral activity (aviD), which is preferentially expressed by nondifferentiated cells, has been recently reported (*22*). On the other hand, a number of viruses of different viral families—including Picornaviridae—are known to encode viral suppressors of RNAi (VSRs) to counteract RNAi-mediated immunity (*3*, *23*). Infection by viruses with disabled VSRs has been found to trigger viral siRNAs (vsiRNAs) production and antiviral RNAi response, while vsiRNAs are not usually detected in the presence of functional VSRs (*24*–*27*). However, recent research shows that canonical vsiRNAs processed from viral dsRNA-replicative intermediates (dsRNA-vRIs) were produced in IFN-competent suckling mice after wild-type (WT) Nodamura virus infection (*28*) and that WT alphaviruses can trigger vsiRNA production and antiviral RNAi in IFN-competent, differentiated mammalian somatic cells and adult mice (*29*). Here, we provide evidence of Dicer cleavage by the FMDV Leader protease and analyze its impact on infection and small viral RNA (svRNAs) production in swine host cells. Our findings reveal unknown features of the intricate interplay between RNA viruses and Dicer in mammalian cells.

## RESULTS

### The FMDV Leader protease cleaves Dicer during infection

**Fig. 1.**
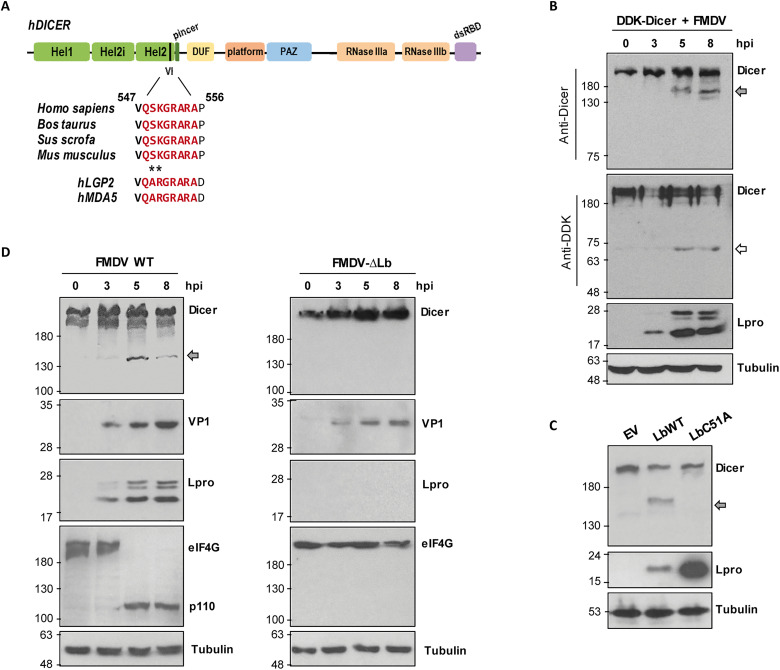
Dicer is cleaved during FMDV infection by Lpro. (**A**) Schematic representation of Dicer showing the putative Lpro target motif conserved across different species including FMDV hosts. (**B**) IBRS-2 cells (1 × 10^6^) were transfected with 2 μg of DDK-Dicer plasmid and 24 hours later infected with FMDV (O1BFS isolate) at an MOI of 5. Cells were lysed at different times after transfection and analyzed by immunoblot for detection of the indicated proteins. (**C**) HEK293 cells (1 × 10^6^) were transfected with 1 μg of plasmids encoding LbWT, LbC51A or an empty vector (EV) and lysed 24 hours later for analysis of the indicated proteins by immunoblot. (**D**) IBRS-2 cells were infected with FMDV WT or FMDV-∆Lb at an MOI of 5 and lysed at different times after infection for immunoblot analysis. The N-terminal and C-terminal cleavage products of Dicer are indicated by white and gray arrows, respectively. The 110-kDa cleavage product of eIF4G is also depicted. Data shown are representative of independent biological replicates (*n* = 2 to 4).

In a search for potential Lpro targets among host antiviral factors, a conserved domain resembling the Lpro cleavage sequence defined in the RIG-I–like receptors LGP2 and MDA5 was identified in the N-terminal Hel2 domain of Dicer. This sequence was conserved in Dicer orthologs across multiple species including humans, mice, and FMDV-susceptible animals such as pigs and cows (Fig. 1A). To test whether Dicer was susceptible to cleavage by Lpro, IBRS-2 swine cells overexpressing the DDK-tagged human sequence of the protein were infected with FMDV. When lysates from infected cells were analyzed by immunoblot, a C-terminal Dicer-derived cleavage product of about 155 kDa was observed from 5 hours onward after infection. Consistently, an N-terminal fragment bearing the DDK tag of 63 to 75 kDa could also be detected at the same time points (Fig. 1B). Next, we tested the integrity of endogenous human Dicer in human embryonic kidney (HEK) 293 cells overexpressing either an active Lbpro (LbWT) or a catalytically inactive mutant of the protease (LbC51A) (*30*). The C-terminal 155-kDa fragment could be readily detected in LbWT-expressing cells unlike those expressing the LbC51A inactive protease (Fig. 1C). We next evaluated whether endogenous swine Dicer was being processed during FMDV infection. For that, IBRS-2 cells were infected with FMDV at a multiplicity of infection (MOI) of 5 and lysed at different times after infection. Again, a Dicer product of about 155 kDa was observed 5 hours onward after infection, supporting the hypothesis that Dicer is cleaved by Lpro during FMDV infection (Fig. 1D). To precisely associate Lpro activity with Dicer degradation, an FMDV mutant lacking the Lb-coding gene (FMDV-∆Lb) (*21*) was assayed. In this case, no sign of Dicer processing was observed up to 8 hours post-infection (pi), while cleavage was detected 3 hours earlier for the WT virus (Fig. 1D). During infection with both viruses the integrity of eIF4G, a known target for Lpro cleavage (*31*) was monitored. As observed for Dicer, eIF4G cleavage was detected at 5 hours pi, while no degradation was detected over FMDV-∆Lb infection (Fig. 1D). Together, these results demonstrate that endogenous Dicer is cleaved in an Lpro-dependent manner during FMDV infection in swine cells.

**Fig. 2.**
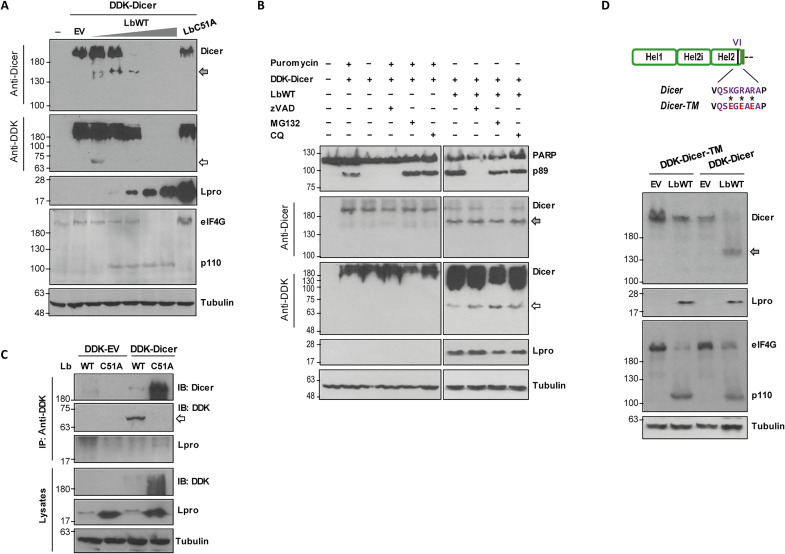
Lpro specifically cleaves Dicer at the KGRAR motif. (**A**) HEK293 cells were cotransfected with 2 μg of DDK-Dicer plasmid and increasing amounts of LbWT (0.2, 2, 20, 200, and 2000 ng), 1 μg of EV, or 1 μg of LbC51A plasmids. Cells were lysed 24 hours later for detection of the indicated proteins by immunoblot. (**B**) HEK293 cells were cotransfected with 2 μg of DDK-Dicer and 300 ng of LbWT plasmids in the presence of zVAD (20 μM), MG132 (10 μM), or chloroquine (CQ) (50 μM). In control cells, apoptosis was induced with puromycin (20 μM). Cells were lysed 24 hours later and analyzed by immunoblot for detection of the indicated proteins using the specified antibodies. (**C**) HEK293 cells were cotransfected with 1 μg of DDK-Dicer or DDK-EV and 1 μg of LbWT or LbC51A plasmids. Cells were lysed 24 hours later, and IP was performed using an anti-DDK monoclonal antibody. IP fractions and lysates were analyzed by immunoblot. (**D**) HEK293 cells were cotransfected with 1 μg of DDK-Dicer or DDK-Dicer-TM (K550E, R552E, and R554E) and 20 ng of LbWT plasmids. Lysates were collected 24 hours later and analyzed by immunoblot. The specific residues modified in DDK-Dicer are shown. The N-terminal and C-terminal cleavage products of Dicer are indicated by white and gray arrows, respectively. The 110-kDa cleavage product of eIF4G is also depicted. Data shown are representative of independent biological replicates (*n* = 2 to 4).

Next, we sought to accurately correlate this cleavage event with the specific enzymatic activity of Lpro. As shown in Fig. 2A, increasing doses of Lb coexpressed with Dicer in HEK293 cells resulted in decreasing levels of Dicer. The N- and C-terminal Dicer cleavage products were detected in coexpression with as little as 0.2 ng of Lb. The activity of Lb on eIF4G was also monitored as control and, as it was observed during FMDV infection, seemed to broadly overlap with Dicer degradation (Fig. 2A). To assess whether the caspase, proteasome, or lysosomal pathways were involved in the degradation of Dicer observed during FMDV infection, Dicer and Lb were coexpressed in HEK293 cells in the presence of the inhibitory compounds zVAD, MG132, or chloroquine, respectively. As shown in Fig. 2B, induction of apoptosis with puromycin did not result in Dicer degradation neither the presence of the inhibitors prevented Dicer cleavage, yielding the previously observed 63-kDa N-terminal product. When the interaction between Dicer and Lpro was analyzed by coimmunoprecipitation (co-IP) assays in HEK293 cells, a faint but clear band could be detected with the anti-Lpro antibody only when Dicer was coexpressed with LbC51A, likely due to the high extent of cleavage observed in lysates from cells coexpressing Dicer and the catalytically active form of Lpro (Fig. 2C). To determine whether the FMDV Lpro was cleaving Dicer at the KGRAR motif, as suggested by sequence analysis and fragment sizes observed (Fig. 1, A to D), a mutant Dicer (Dicer-TM) in which the positively charged K/R amino acids were replaced by negatively charged E (K550E, R552E, and R554E) was generated to abolish Lpro cleavage as previously shown for LGP2 and MDA5 (*18*, *19*). Unlike Dicer-WT, when Dicer-TM was coexpressed with LbWT, no cleavage fragments were observed, although eIF4G degradation was complete (Fig. 2D). As a whole, these results demonstrate that Dicer is cleaved during FMDV infection by the Leader protease at the KGRAR motif.

### Dicer restricts the replication of an Lpro-deficient FMDV mutant

**Fig. 3.**
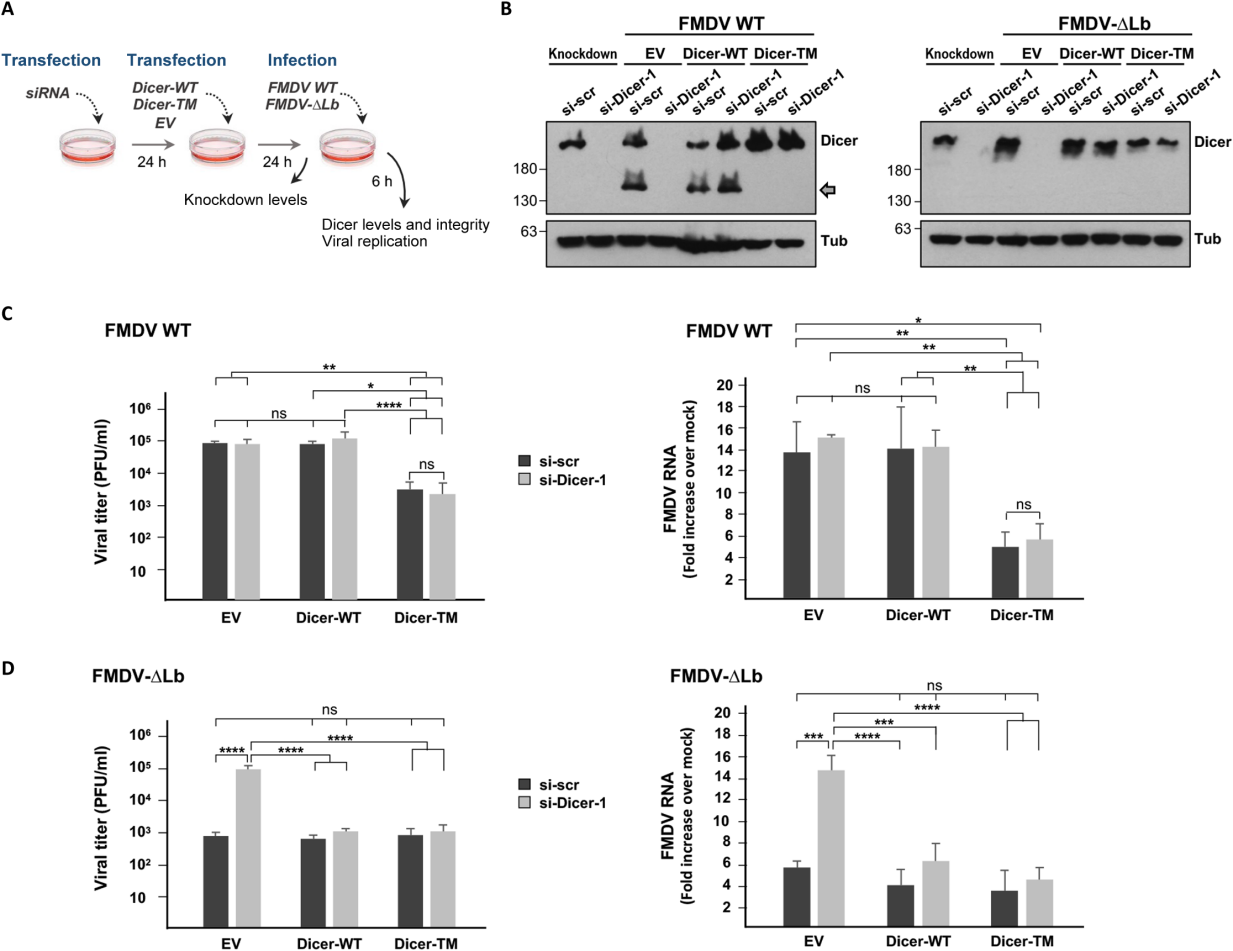
The ability of FMDV to cleave Dicer correlates with replication efficiency. (**A**) IBRS-2 cells were transfected with 100 nM specific Dicer siRNAs (si-Dicer-1) or a scramble siRNA and 24 hours later transfected with 500 ng of DDK-Dicer, DDK-Dicer-TM or an EV. After 24 hours, cells were infected with FMDV WT or FMDV-∆Lb at an MOI of 5. At 6 hours after infection, supernatants were collected and cells lysed for protein analysis and RNA extraction. (**B**) Western blot analysis of Dicer levels and integrity at 6 hours after infection. The knockdown efficiency prior to infection was analyzed 24 hours after transfection with the EV (knockdown lanes). The C-terminal cleavage product of Dicer is indicated by a gray arrow. (**C** to **D**) Virus titration of supernatants by plaque assay in BHK-21 cells and quantification of viral RNA by RT-qPCR of cells infected with FMDV WT (C) or FMDV-∆Lb (D). Data shown in (C) and (D) are means ± SD of three independent experiments (*n* = 3). Statistical analyses were performed by one-way ANOVA test with Tukey’s correction. *P < 0.05, **P < 0.01, ***P < 0.001, and ****P < 0.0001; ns, not significant. Representative results from a single experiment are shown in (B).

To assess the impact of Dicer during FMDV infection in swine cells, its endogenous expression was silenced by RNAi. For that, IBRS-2 cells were transfected with a Dicer-specific siRNA (si-Dicer-1) or a scrambled control siRNA. Cells were then transfected with plasmids expressing Dicer-WT or Dicer-TM proteins and further infected at an MOI of 5 with either FMDV WT or FMDV-∆Lb (deficient for Lpro) for 6 hours (Fig. 3A). As shown in Fig. 3B, Dicer was specifically silenced and its levels restored after overexpression of both Dicer-WT and Dicer-TM, unlike after transfection with an empty vector (EV). After 6 hours of infection, the C-terminal cleavage product could be detected only in cells infected with FMDV WT and expressing Dicer-WT or in those transfected with the scramble siRNA and the EV (endogenous Dicer). When viral progeny was quantified in the supernatants, no significant differences in viral titers could be observed between cells infected with FMDV WT transfected with Dicer-specific or with control siRNAs and the EV (Fig. 3C). In contrast, Dicer silencing resulted in an enhanced replication of FMDV-∆Lb (Fig. 3D). Consistently, a substantial increase in the accumulation of viral RNA was detected in IBRS-2 cells infected with FMDV-∆Lb, when pretreated with the Dicer-specific siRNAs compared with control siRNA (Fig. 3D). The overexpression of Dicer before infection had a different impact on viral replication for the two viruses depending on whether the WT or the noncleavable version of Dicer (Dicer-TM) was expressed. While Dicer-WT expression did not affect FMDV WT titers significantly, Dicer-TM expression was associated with a decrease in viral titers and intracellular viral RNA regardless of the siRNA initially transfected (Fig. 3C). In contrast, when Dicer levels were restored by expression of either Dicer version before infection with FMDV-∆Lb, similar titers and RNA levels to those in cells transfected with the scramble siRNA and the EV were observed (Fig. 3D). These results show a clear correlation between the ability of FMDV to cleave Dicer and the viral replication levels in host cells. The impact of silencing Dicer in FMDV WT and FMDV-∆Lb titers and RNA levels was further confirmed using a second siRNA targeting a different Dicer sequence (si-Dicer-2) or a mixture of them (si-Dicer-1 + 2) after infection for 2 or 4 hours (fig. S1). Furthermore, no relevant effect on the integrity and levels of eIF4G and G3BP1—two known Lpro targets actively involved in antiviral defense—could be detected in cells transfected with the Dicer-specific siRNAs compared with scrambled siRNA at the time of infection (fig. S1). Collectively, these findings strongly suggest that Dicer is a restriction factor for FMDV infection, which is effectively counteracted by the Lpro catalytic activity.

### Lpro impairs shRNA-induced RNAi in mammalian cells

**Fig. 4.**
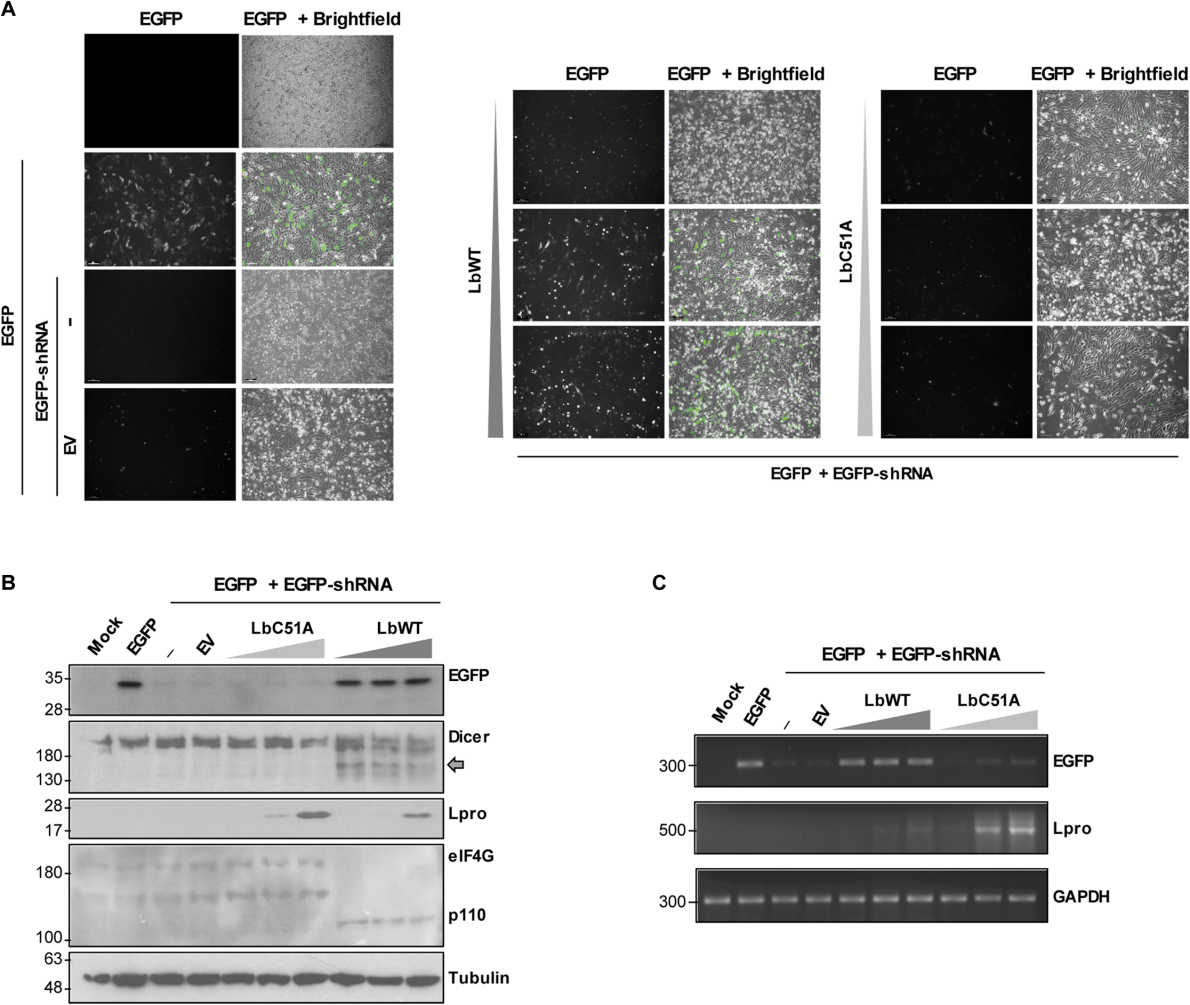
FMDV Lpro suppresses the shRNA-induced RNAi mediated by Dicer in swine cells. (**A** to **C**) IBRS-2 cells were cotransfected with plasmids encoding EGFP (0.3 μg) and a specific EGFP shRNA (5 μg) together with an EV (100 ng) or a plasmid encoding LbWT or LbC51A (0.2, 2, or 20 ng). At 48 hours after transfection, cells were directly observed by fluorescence and bright-field microscopy and images were taken with a ×10 magnification (A) and then lysed for detection of the indicated proteins by Western blot (B); the C-terminal cleavage product of Dicer is indicated by a gray arrow. The levels of EGFP, Lpro, and GAPDH mRNAs were examined by RT-PCR after RNA extraction from the same lysates (C). Data shown are representative of independent biological replicates (*n* = 2 to 4).

**Fig. 5.**
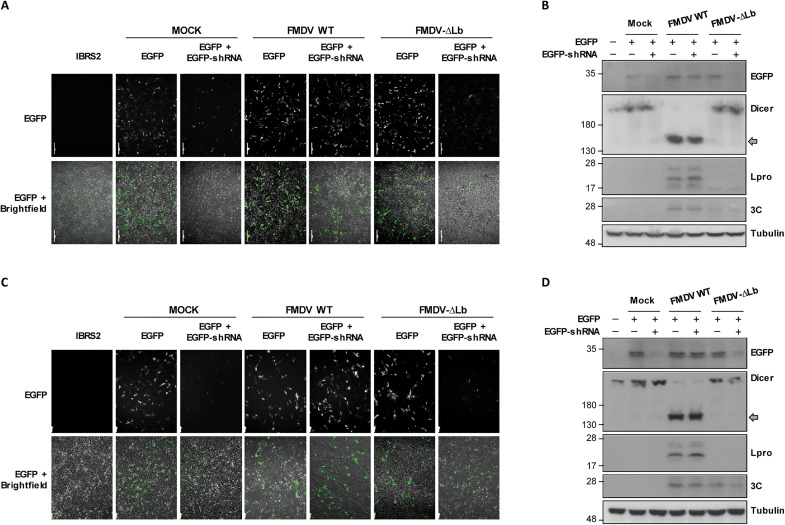
Deletion of Lbpro impairs the suppression of shRNA-induced RNAi phenotype of FMDV in swine cells. IBRS-2 cells were cotransfected with plasmids encoding EGFP (0.3 μg) and a specific EGFP shRNA (5 μg) for 24 hours and then mock-infected or infected with FMDV WT or FMDV-∆Lb at an MOI of 0.001 (**A**) or an MOI of 1 (**C**). EGFP fluorescence and bright-field microscopy images were taken at 20 hours (A) or 12 hours (C) after infection with a ×10 magnification and then lysed for detection of EGFP, Dicer, FMDV Lpro, FMDV 3C, or tubulin by Western blot (**B** and **D**). All results are representative of at least two independent experiments.

Having shown that Dicer is a target for the FMDV Lpro, we examined whether Lpro had RNAi-suppressing activity. For that, a reversal-of-silencing assay was performed by cotransfection of swine IBRS-2 cells with plasmids encoding enhanced green fluorescent protein (EGFP) and an EGFP-specific small hairpin RNA (shRNA), which is cleaved by Dicer to generate siRNA targeting EGFP, together with either an active Lbpro (LbWT) or an inactive mutant Lb (LbC51A). After 48 hours of transfection, the levels of EGFP expression were analyzed by fluorescence microscopy and Western blot, and the EGFP mRNA levels were determined by reverse transcription polymerase chain reaction (RT-PCR) (Fig. 4, A to C). The expression of the EGFP shRNA eliminated fluorescence signal (Fig. 4A) and detectable protein (Fig. 4B), and only very low levels of EGFP mRNA were detected (Fig. 4C), indicating that RNAi could be efficiently induced in IBRS-2 cells by shRNA. Very similar results on EGFP expression were obtained when LbC51A was expressed. However, LbWT expression effectively rescued the EGFP fluorescence, protein, and mRNA levels even at the lowest amount assayed (0.2 ng), which was below the detection limit by Western blot (Fig. 4, A to C). The catalytic activity of Lbpro was monitored by eIF4G cleavage (Fig. 4B). The Lbpro-dependent cleavage of Dicer was evident at all three amounts assayed, while cotransfection of 100 ng of EV did not affect shRNA-induced EGFP silencing or Dicer integrity (Fig. 4B). In the absence of EGFP shRNA, no effect on EGFP fluorescence intensity was observed at 0.2 and 2 ng of LbWT, while the expression of 20 ng reduced EGFP signal likely due to its negative impact on translation (fig. S2). To test whether the different reversal-of-silencing phenotypes exhibited by LbWT and LbC51A could be reproduced in the context of infection by FMDV WT and FMDV-∆Lb viruses, respectively, IBRS-2 cells expressing EGFP and EGFP shRNA were infected 24 hours later with each virus at low or high MOI (Fig. 5, A to D). While EGFP fluorescence was effectively rescued in FMDV WT–infected cells at both viral doses, in cells infected with FMDV-∆Lb, the reversal mechanism was impaired (Fig. 5, A and C). The Western blot analysis of EGFP, Dicer, and FMDV-encoded Lpro and 3C proteins was consistent with the fluorescence observed and the corresponding viral genotype and dose used (Fig. 5, B and D).

**Fig. 6.**
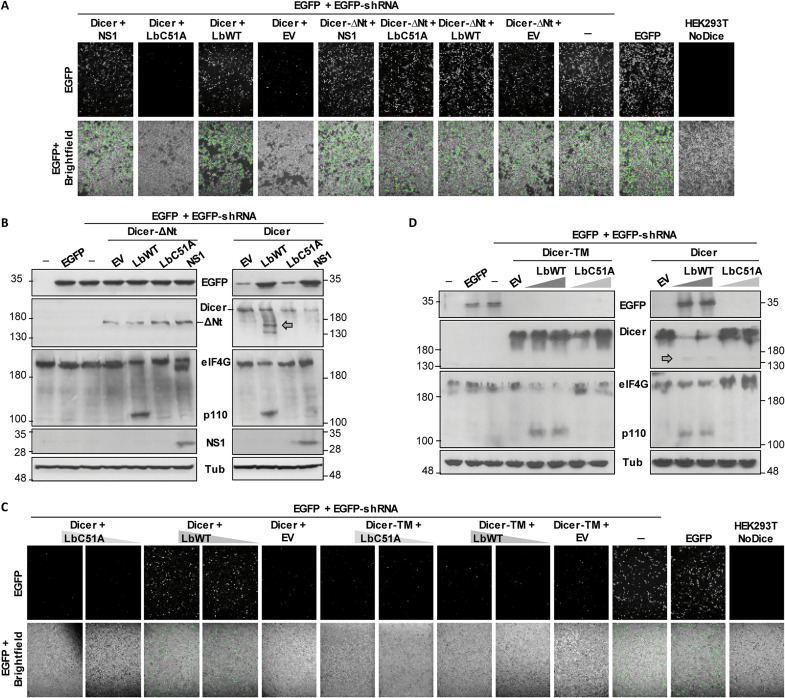
Analysis of the shRNA-induced RNAi mediated by Dicer-∆Nt and Dicer-TM in HEK293T NoDice cells. (**A** and **B**) HEK293T NoDice cells were cotransfected with plasmids encoding EGFP (0.1 μg) and a specific EGFP shRNA (5 μg) together with 0.5 μg of plasmids encoding Dicer or Dicer-∆Nt, LbWT (2 ng), LbC51A (2 ng), or IAV NS1 (0.3 μg), and EGFP fluorescence and bright-field microscopy images were taken at 24 hours after transfection with a ×10 magnification (A). Cells were then lysed for protein analysis by Western blot (B). (**C** and **D**) HEK293T NoDice cells were cotransfected with plasmids encoding EGFP (0.1 μg); EGFP shRNA (5 μg) together with 0.5 μg of a plasmid encoding Dicer or Dicer-TM and LbWT, LbC51A (0.2 or 2 ng); or EV (2 ng). EGFP fluorescence and bright-field microscopy images were taken at 24 hours after transfection with a ×10 magnification (C), and cells were then lysed for detection of the indicated proteins by Western blot (D). All results are representative of at least two independent experiments.

Next, we sought to address the RNAi activity of a Dicer mutant resembling the resulting protein after Lpro cleavage. For that, a construct lacking the 555 N-terminal amino acids comprising almost the complete helicase domain was generated (Dicer-∆Nt) and assayed for EGFP silencing in *Dicer* knockout HEK293T cells (NoDice) (*32*). As shown in Fig. 6A, the expression of Dicer-∆Nt—unlike full-length Dicer—was unable to rescue the shRNA-induced EGFP silencing in NoDice cells. Consistent with this, the expression of an active Lbpro rescued EGFP fluorescence signal in NoDice cells also expressing Dicer but not in those expressing Dicer-∆Nt. When the nonstructural protein NS1 of influenza A virus, known to suppress RNAi (*25*), was coexpressed with each Dicer form, equivalent results to those with Lpro were observed (Fig. 6A). The EGFP protein levels detected by Western blot were consistent with the intensity of fluorescence in all cases, and Dicer cleavage was only detected in NoDice cells expressing Lbpro and full-length Dicer (Fig. 6B).

To further associate the Dicer cleavage event with its impaired capacity for shRNA-induced silencing, the noncleavable mutant Dicer-TM was tested in the reversal-of-silencing assay. Dicer-TM was fully functional for EGFP silencing in NoDice cells (Fig. 6C), but its coexpression with Lbpro did not affect fluorescence intensity or EGFP levels detected by Western blot (Fig. 6, C and D). Together, these results indicate that Dicer cleavage by Lpro at the KGRAR motif abrogates RNAi induced by shRNA in mammalian cells.

### Impact of Lbpro depletion on viral small RNAs production during FMDV infection

Having proven the impact of Dicer cleavage by Lpro on FMDV infection and shRNA-induced RNAi, we further examined the production and profiles of virus-derived small RNAs in swine cells infected with FMDV WT or FMDV-∆Lb. For that, RNA from IBRS-2 cells infected with each virus at an MOI of 5 was extracted at 4 hours pi and subjected to deep sequencing. The number of reads aligned to the FMDV genome was 4.5-fold lower for FMDV-∆Lb compared to FMDV WT (table S1) and consistent with the lower replication levels measured for FMDV-∆Lb in IBRS-2, as shown in fig. S3.

**Fig. 7.**
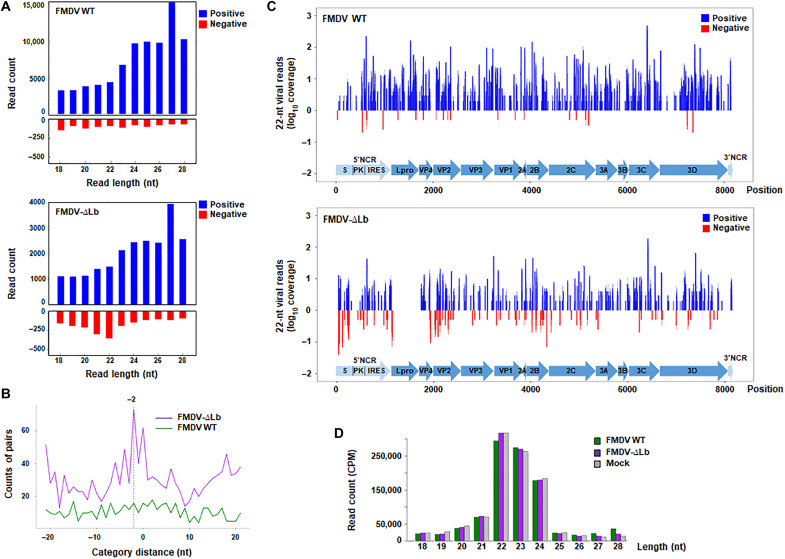
Production of FMDV-derived small RNAs in swine cells. IBRS-2 cells were infected with FMDV WT or FMDV-∆Lb at an MOI of 5 or mock-infected. RNA was extracted at 4 hours pi and subjected to deep sequencing. (**A**) Size distribution and abundance of vsRNAs (positive-stranded in blue and negative-stranded in red) shown as counts mapped to the viral genome. (**B**) Presence of pairs of 22-nt reads with 2-nt 3′-overhangs (−2 peak), defined as canonical vsiRNAs. (**C**) Distribution of 21- to 23-nt vsRNAs in the FMDV WT and FMDV-∆Lb genomes. The relative abundances of positive- and negative-stranded vsRNAs are indicated (coverage per nucleotide of the viral genome). The different proteins encoded by the FMDV RNA and the flanking 5′ and 3′ NCRs are shown with arrows. (**D**) Abundance of 18- to 28-nt RNAs (cellular and viral small RNAs) in IBRS-2 cells infected with FMDV WT–, FMDV-∆Lb– or mock-infected shown as counts per million of total reads (CPM). Data represent one independent experiment.

In RNAi, canonical siRNAs resulting from Dicer activity are generated as 22 ± 1–nt RNA duplexes with 3′2-nt overhangs. As illustrated in Fig. 7A, the vsRNA population in IBRS-2 cells infected with FMDV-∆Lb was enriched for the 22-nt negative strand vsRNAs unlike cells infected with FMDV WT, which showed a nonclustered pattern. When the abundance of perfectly base-paired 20-nt duplexes with 2-nt 3’overhangs was analyzed, a clear “peak −2” could be detected for the 22-nt vsRNAs produced in FMDV-∆Lb–infected cells, which was not found for the vsRNAs of FMDV WT (Fig. 7B). Relevant differences in the abundance and distribution of vsRNAs along the FMDV genome could be observed also comparing the two viruses (Fig. 7C and table S1). A relevant enrichment in 22-nt reads of negative polarity was observed for FMDV-∆Lb (18.9% versus 1.7%), while the 22-nt vsRNAs detected for FMDV WT were predominantly positive stranded (98.2%) and distributed along the genome (Fig. 7C and table S1). This enrichment was mainly detected in the 5´-terminal S-fragment region revealing apparent peaks of vsRNAs of both polarities (Fig. 7C). A similar result was described for the vsiRNAs generated during infection with a VSR-deficient dengue virus type 2 (DENV2) virus. In this case, the vsiRNAs of negative polarity clustered in both the 5′- and the 3′-terminal regions of the viral genome (*24*)*.* For FMDV-∆Lb, no accumulation of (−)vsRNAs in the 3′ terminus was observed, whereas there seemed to be an enrichment in other specific regions like the AUG1/2, VP4, VP2, and 2B (Fig. 7C). Comparison of the length distribution of total reads for FMDV WT–, FMDV-∆Lb–, and mock-infected IRBS2 cells showed a lower level of 22-nt small RNAs in cells infected with FMDV WT, while their abundance in FMDV-∆Lb– and mock-infected cells was very similar and slightly higher (Fig. 7D), suggesting that Dicer cleavage by Lpro may affect also the production of host miRNAs.

## DISCUSSION

Viruses have evolved different mechanisms to counteract host defenses aimed at ensuring replication and cell-to-cell propagation. Suppression of the RLR signaling pathway and the downstream IFN-I signaling pathway is a common strategy used by viruses to dampen the host antiviral response by targeting relevant sensors or signaling molecules for direct cleavage or inducing their degradation (*33*). The IFN system is absent from invertebrates and plants, which use RNAi to protect themselves from viral infection (*3*). Although mammalian cells express all the components for RNAi, the role of this pathway as a functional antiviral mechanism has been controversial (*3*, *34*). Recent research suggests that the activity of virally encoded VSRs might have masked the detection of vsiRNAs generated during infection. Using more refined protocols before deep sequencing such as removing nonspecific small RNAs or examining in vivo infection at different time points may facilitate detection of vsiRNAs during infection with WT viruses (*28*).

Most viral proteins displaying VSR activity in mammalian cells are dsRNA binding proteins that act sequestrating dsRNA from Dicer, while other VSRs bind to Dicer or its cofactors to inhibit Dicer activity (*3*). Adenoviruses generate small highly structured RNAs that inhibit RNAi by acting as decoy RNA substrates for Dicer (*35*, *36*). The role of noncoding subgenomic flavivirus RNA (sfRNA) as RNAi suppressors by their interaction with Dicer has also been suggested (*37*, *38*). Here, we showed that Dicer expression restricts FMDV infection, while the virus-encoded Lpro cleaves Dicer releasing the complete helicase domain. Our results strongly suggest that FMDV is using Dicer cleavage by Lpro to evade the antiviral effect exerted by Dicer by means of a strategy which is intrinsically different to those used by all VSRs described to date.

Deep sequencing analysis revealed different production and profiles of virus-derived small RNAs in swine cells infected with FMDV WT or with an Lpro-deficient virus (FMDV-∆Lb). An increase in 22-nt reads of negative polarity was observed for FMDV-∆Lb together with an enrichment in duplexes meeting the requirements of canonical siRNAs resulting from Dicer activity. This analysis was performed in swine kidney epithelial IBRS-2 cells. In IBRS-2 cells, the RLR signaling pathway seems to be impaired due to a noncharacterized defect in signal transduction at the TBK1/IRF3 level. However, IBRS-2 cells have an intact Janus kinase/signal transducers and activators of transcription signaling pathway and a reduced but measurable ISG induction was observed upon transfection with the dsRNA analog polyinosinic:polycytidylic acid [poly(I:C)] (*39*). The crucial role of MDA5 in sensing FMDV infection has been documented, as the lack of MDA5-dependent IFN induction in FMDV-infected IBRS-2 cells (*18*). In this context, the absence of RLR-triggered IFN response during FMDV infection is not expected to mask any RNAi-dependent antiviral effect that might be operating in IBRS-2 cells. Moreover, FMDV-∆Lb grows poorly in cells capable of an IFN-α/β response, hampering the parallel analysis in fully IFN-competent swine cells. Nevertheless, future efforts will be directed to generate swine cell lines knockout for different Lpro targets that would allow to assess their individual contribution to impair FMDV infection in the presence or absence of an active IFN response.

Whether the anti-FMDV activity of Dicer is fully or partly dependent on RNAi or else relies on other noncanonical mechanisms will need further research. Also, other roles involving miRNAs biogenesis potentially interfering with the miRNA pathway and affecting cellular gene expression to promote viral replication cannot be ruled out. Addressing if pre-miRNA maturation is disrupted in infected cells when Dicer is being cleaved by Lpro is a relevant issue that might be the subject of further work.

Beyond its crucial role in RNAi, the involvement of Dicer in non-RNAi–related signaling pathways is being increasingly unveiled and the cross-talk between different antiviral pathways operating in mammalian cells (*40*, *41*). The ability of specific positive-sense vsRNAs from the enterovirus 71 (EV71) IRES stem-loop II to reduce IRES activity and viral replication has been reported (*42*). These vsRNAs are generated by Dicer cleavage on the highly structured 5′ untranslated region in the viral genome.

The N-terminal helicase domain of Dicer is composed by three subdomains (Hel1, Hel2i, or Hel2) and has been proposed to act as a platform for the recruitment of different proteins to diversify the functions of DICER (*43*, *44*). This domain is known to interact with several dsRNA binding proteins and RNA helicases during viral infection including the TAR-RNA binding protein (TRBP) (*45*), the protein activator of interferon-induced protein kinase R (PACT) (*46*), and the protein kinase RNA-activated (PKR) (*43*). Interaction with these co-factors is needed to ensure the Dicer full functionality. The reported ability of the helicase domain alone to bind PKR, TRBP, and PACT (*43*) raises the possibility that FMDV might be using the N-terminal fragment of Dicer generated by Lpro cleavage and comprising nearly the entire helicase domain as a decoy to sequester these factors to hamper or modulate the antiviral activity of PKR and the proper functioning of Dicer.

Truncated Dicer proteins with deletions in the helicase domain have been shown to display an antiviral phenotype against infection by several viruses (*47*). This differential function has been recently associated with the disrupted interaction of the mutants with PKR, which through a noncatalytic NF-κB–dependent manner would modulate the inflammatory response with different results depending on the particular virus (*48*). A proviral effect was associated with the expression of the N1 mutant lacking the first two subdomains (∆Hel1 and ∆Hel2i) (*47*) in the context of severe acute respiratory syndrome coronavirus 2 infection. The antiviral phenotype of the Dicer mutants carrying individual deletions of each subdomain seemed to rely on RNAi unlike that of the N1 mutant. The antiviral effect of all mutants, including those resembling the AviD isoform expressed in human embryonic stem cells (∆Hel2i) (*22*) and the retrotransposon-driven Dicer^o^ isoform expressed in mouse oocytes (∆Hel1) (*49*), depended on the presence of PKR (*48*).

The silencing of Dicer in swine cells selectively promoted the replication of FMDV-∆Lb unlike the WT virus. Moreover, this replication-enhancing effect could be reverted by replacement of Dicer before infection regardless of whether the WT or the noncleavable form of the protein was expressed. In contrast, the levels of replication of FMDV WT were significantly reduced only when the noncleavable version of Dicer was expressed, revealing that the ability of FMDV to cleave Dicer by the Lpro catalytic activity has a positive impact on viral fitness. We did not detect any difference in the enhancing effect exerted by the two siRNAs assayed. The si-Dicer-2 targets an RNA sequence in Hel2i that is absent in AviD, suggesting that the contribution of this Dicer isoform with enhanced antiviral activity to the Dicer-dependent anti-FMDV effect observed in IBRS-2 cells is not significant. This is consistent with the low levels of AviD expression found in differentiated cells (*22*).

A Dicer mutant lacking the entire helicase domain, named Dicer N3, exceeding in 52 amino acids the size of the protein fragment removed by Lpro, exhibited a reduced capacity for processing miRNAs (*47*), in agreement with our results showing the inability of the Lpro-truncated Dicer (Dicer-∆Nt) to rescue the shRNA-induced EGFP silencing in NoDice cells.

The catalytic activity of the FMDV Lpro impairs a variety of immune effectors from different signaling routes involved in the host antiviral response against infection. Dicer and the RLR family members LGP2 and MDA5 are cleaved by Lpro at an equivalent DExD/H-box helicase motif (K/RGRAR), evidencing that Lpro targets domains conserved during evolution in RNA binding proteins of relevance for viral growth optimizing the small size of the FMDV genome to encode multifunctional proteins with pleiotropic effects against host defenses. The fact that these helicase domains targeted by Lpro are also conserved among different host species for the virus, as found for Dicer, might also contribute to the broad host range of FMDV compared with other picornaviruses lacking a Leader protease. LGP2 is actively involved in the IFN-mediated inhibition of antiviral RNAi in differentiated mammalian cells through direct interaction with Dicer blocking processing of long dsRNA and siRNA production (*22*, *28*, *50*) and with TRBP repressing specific miRNA activities (*51*). The LGP2-dependent modulation of RNAi illustrates the cross-talk between RNAi and other innate immune pathways. It seems plausible that viruses may have evolved strategies to counteract different antiviral pathways taking advantage of having their effector proteins in close vicinity, as found for the FMDV Lpro that cleaves both LGP2 and Dicer resulting in enhanced viral replication in infected cells. Learning how viruses interact with the immune system to circumvent the host defenses is crucial for understanding the molecular basis of infection and developing antiviral strategies.

## MATERIALS AND METHODS

### Cells and viruses

Swine kidney IBRS-2 cells were obtained from Centro de Investigación en Sanidad Animal, CISA-INIA-CSIC, Spain). Human kidney HEK293 and baby hamster kidney (BHK)–21 cells were originally sourced from the American Type Culture Collection. HEK293T/NoDice and parental HEK293T cell lines were a gift from B. Cullen (Duke University School of Medicine, USA) and have been previously described (*32*). All cell lines were grown in Dulbecco’s modified Eagle’s medium (DMEM) (Gibco) supplemented with 10% fetal bovine serum (FBS), penicillin/streptomycin, and 1% l-glutamine at 37°C and 5% CO_2_. FMDV O1BFS isolate was obtained from the Centro de Investigación en Sanidad Animal (CISA-INIA-CSIC, Spain). FMDV WT and FMDV-ΔLb viruses derive from an FMDV O1K full-length cDNA clone and have been described previously (*21*, *52*).

### Transfections and infections

Subconfluent monolayers of the different cell lines seeded 24 hours before were transfected with plasmids (0.2 to 5 μg) using Lipofectamine 2000 (Invitrogen) and Opti-MEM I reduced serum medium (Gibco) following the manufacturer’s instructions. In some transfection experiments, 20 μM puromycin (Sigma-Aldrich), 20 μM zVAD-FMK (Promega), 50 μM chloroquine (Sigma-Aldrich), or 10 μM MG132 (Cayman Chemical) was added to the transfection medium. For virus infection, cell monolayers (about 1 × 10^6^) were washed and incubated for 1 hour at 37°C with the virus diluted in DMEM without FBS at the MOI indicated in the figure legends. Then, the viral inoculum was removed, and cells were washed twice with DMEM and further incubated with fresh DMEM supplemented with 10% FSB, l-glutamine, and penicillin/streptomycin and incubated at 37°C and 5% CO_2_. At the indicated time after infection, supernatants were collected and viral titers were determined by plaque assay and expressed as plaque-forming units/ml. Cells were lysed and processed for RNA or protein analysis.

### DNA constructs

A plasmid encoding the human Dicer sequence with an N-terminal DDK tag (DDK-Dicer) was a gift from B. TenOever (New York University, USA). The DDK-Dicer-TM construct, designed to replace the three positively charged arginine (R) residues by negatively charged glutamic acid (E) residues (KGRARA/EGEAEA) in the conserved motif VI of the helicase domain, was generated by overlapping recombinant PCR using DDK-Dicer as a template and primers DicerForwExt (5′-GAGCAAGAGGAGCTGCACAGGAAA-3′), DicerTMAntisense (5′-ATTAGAGATGGGTGCCTCTGCTTCTCCTTCAGATTGAACATAGGATCGATATTCTGTGGGC-3′) and DicerTMSense (5′-GCCCACAGAATATCGATCCTATGTTCAATCTGAAGGAGAAGCAGAGGCACCCATCTCTAAT-3′), and DicerRevExt (5′-AGACTTCTTCAACTCAATGGATATGGT AACC -3′). The resulting PCR fragment was digested with Dra III and Bst II and last cloned into DDK-DICER at the Dra III and Bst II restriction sites. The DDK-Dicer-ΔNt construct has a deletion of nucleotides 1 to 1659 from the initiation codon of DDK-Dicer sequence and was generated by recombinant PCR using DDK-Dicer as a template and primers CleavedDicerForw (5′-GAGGCGATCGCCAGGGCACCCATCTCTAATTATAT-3′) and CleavedDicerRev (5′-GCGACGCGTGCTATTGGGAACCTGAGGTTGATT-3′), which introduced flanking Sgr I and Mlu I restriction sites (underlined). The PCR product was then digested with Sgr I and Mlu I and cloned into pCMV6-AN-Myc-DDK (PS100016, OriGene) at the Sgr I and Mlu Irestriction sites. Plasmids encoding the WT protease (LbWT) or the catalytically inactive mutant (LbC51A) of FMDV have been previously described (*19*). The NS1-IAV plasmid expressing the WT NS1 protein of influenza A virus and DDK-CAGGS were both provided by A. García-Sastre (Icahn School of Medicine at Mount Sinai, NY, USA). The EGFP-C1 plasmid (6084-1, Clontech) encodes a red-shifted variant of WT GFP. The shRNA-EGFP plasmid (TR30001, OriGene) expresses an shRNA-EGFP cloned into the pRS retroviral vector using the U6 promoter. The cDNA3.1(+) plasmid (purchased from Invitrogen) and DDK-CAGGS plasmid were used as EV controls in the corresponding transfection experiments. The FMDV O1K-ΔLb construct bearing a deletion of the Lb-coding sequence has been previously described (*21*).

### Antibodies and Western blotting

Transfected or infected cells were collected in lysis buffer (50 mM Tris-HCl [pH, 7.5], 150 mM NaCl, 0.5% NP - 40) supplemented with protease inhibitor cocktail (Complete, Roche) and then clarified by centrifugation at 9.300*g* for 5 min at 4°C. The protein concentration was determined by the Bradford method and cell lysates were subjected to 6–12% SDS-polyacrylamide gel electrophoresis, transferred to nitrocellulose membranes and blocked by PBS containing 3% skimmed milk and 0.05% Tween20 for 90 min at room temperature. The membranes were then probed by incubation with specific antibodies against Dicer (ab227518, Abcam), FLAG (F1804 and F7525, Sigma-Aldrich), the FMDV Leader protease (raised against the Lab/Lb fusion protein and kindly provided by E. Beck, Justus-Liebig University, Giessen, Germany), eIF4G (sc-9602, Santa Cruz Biotechnology), poly(adenosine 5′-diphosphate–ribose) polymerase (PARP) (9542, Cell Signaling Technology), cleaved PARP (9546, Cell Signaling Technology), GFP (11814460001, Roche), G3BP1 (AB_398438, BD Bioscience), IAV NS1 protein (*53*) (a gift from J. Ortín, CNB, Spain), FMDV 3C (2D2) and FMDV type-O VP1 (B2) (both provided by E. Brocchi, IZSLER, Italy), and β-tubulin (T4026, Sigma-Aldrich) overnight at 4°C. Then, membranes were incubated with the horseradish peroxidase–conjugated secondary antibodies (Thermo Fisher Scientific) at room temperature for 1 hour, and proteins were visualized by enhanced chemiluminesence (PerkinElmer).

### co-IP assay

Interaction between Dicer and FMDV Lb was analyzed by co-IP and Western blot assay. HEK293 cells (1 × 10^6^) seeded in a six-well plate were cotransfected with 1 μg of plasmids Lb-WT or LbC51A together with 2 μg of DDK-Dicer or DDK-EV (DDK-CAGGS). Cells were harvested 24 hours after transfection and lysed in 100 μl of lysis buffer, as above. Protein lysates were clarified by centrifugation at 9.300*g* for 5 min at 4°C, and supernatants precleared with 50 μl of protein G agarose beads (Roche) for 2 hours at 4°C with rotation. Then, IP with 2 μg of monoclonal anti-FLAG M2 antibody (Sigma-Aldrich) was performed as described (*19*). Lysates and IP fractions were analyzed by Western blot.

### EGFP reversal-of-silencing assay

To address the effect of Lb expression on shRNA-induced EGFP silencing, IBRS-2 cells (1 × 10^6^) were cotransfected with 0.3 μg of EGFP-C1 and 5 μg of shRNA-EGFP plasmids, together with different amounts of FMDV LbWT or LbC51A plasmids (0.2, 2, and 20 ng). In the assays performed to assess Dicer functionality in NoDice cells, 0.1 μg of pEGFP-C1 and 5 μg of shRNA-EGFP together with 0.5 μg of DDK-Dicer, DDK-Dicer-TM, or DDK-ΔNt-Dicer plasmids were combined for transfection as indicated in the figure legends. In some experiments, the indicated amounts of a plasmid encoding the influenza A virus NS1 protein or an EV [pcDNA3.1(+)] were transfected as positive and negative controls for the suppression of EGFP RNAi, respectively.

The level of EGFP silencing was analyzed at 24 or 48 hours after transfection in HEK293T or IBRS-2 cells, respectively, by fluorescence microscopy. EGFP expression levels were also monitored by Western blot and RT-PCR. Fluorescence emitted by EGFP expression was detected using a Leica DM IL LED inverted microscope coupled to a Leica DFC3000G charge-coupled device microscope camera (Leica microsystems), and phase contrast and bright-field images of the transfected cells were acquired at ×10 magnification (HI PLAN ×10/0.25 PH1 objective) and captured with Leica Application Suite software version 4.12.0 (Leica Microsystems, CMS GmbH, Switzerland).

For amplification of EGFP, Lpro, and glyceraldehyde-3-phosphate dehydrogenase (GAPDH) mRNAs by RT-PCR, 50, 150, and 500 ng of total RNA, respectively, and the following primers were used: EGFP-Fwd (5′-ACGTAAACGGCCACAAGTTCAG-3′), EGFP-Rev (5′-TTCACCAGGGTGTCGCCC-3′), LbMT-sense (5′-CCAACAACCACGACAACGCCTGGTTGAACGCCATCC-3′), Lb-antisense (5′-GCTAGTCTAGAGTTTGAGCTTGCGTTGAACCTTGG -3′), GAPDH-aei025 (5′-CATCACCATCTTCCAGGAGCGAG-3′), and GAPDH-aei021 (5′-AAGTTGTCATGGATGAC-CTTGGCCA-3′). RT-PCR conditions were as follow: 1 cycle of 30 min at 48°C and 35 cycles of 95°C for 30 s, 55°C for 30 s, and 72°C for 45 s. The size of the EGFP, Lpro, and GAPDH amplicons are 301, 474, and 298 base pair (bp), respectively.

### RNA isolation and RT-qPCR

Cells were collected in lysis buffer containing 50 mM tris-HCl (pH 7.8), 0.5% NP-40, and 120 mM NaCl, and total RNA was extracted with Tri-Reagent (Sigma-Aldrich), treated with deoxyribonuclease (Turbo DNA-free kit, Ambion) and quantified using a Nanodrop ND-1000 (Thermo Fisher Scientific). For quantification of the FMDV RNA by RT-qPCR, 500 ng of total RNA was used in the RT reaction with SuperScript IV Reverse Transcriptase (Invitrogen). Then, quantitative PCR was performed with aliquots of the RT reactions (1/10) using the GoTaq qPCR Master Mix (Promega) in hard-shell 384-well plates and CFX Opus 384 Real-Time PCR System (Bio-Rad). The FMDV 3D oligonucleotides used have been previously described (*54*). The relative abundance of viral RNA was calculated using the ∆∆*C*t method normalizing to GAPDH and was expressed as the fold increase above the siRNA-transfected mock-infected cells.

### Dicer knockdown

Chemically synthesized 21-nt siRNA duplexes were purchased from Dharmacon. siRNA Dicer-1 (5′-GGAAAGAGACGGUUAAAUAUU-3′) targets swine Dicer (corresponding to 2150 to 2168 nt; GenBank, HQ184403.1). Swine scramble siRNAs (scr) (*55*) were used as control. IBRS-2 cells (5 × 10^5^) were transfected with 100 nM siRNAs using Lipofectamine 2000 (Invitrogen) and, 24 hours later, transfected with 500 ng of DDK-Dicer, DDK-Dicer-TM, or an EV. After 24 hours, cells were infected with FMDV WT or FMDV-∆Lb at an MOI of 5 for 6 hours. Then, supernatants and cells were collected for virus titration by plaque assay in BHK-21 cells, quantification of viral RNA by RT-qPCR, and determination of Dicer expression levels by Western blot.

### Deep sequencing and data analysis

IBRS-2 cells were mock-infected or infected with FMDV WT or FMDV-ΔLb at an MOI of 5 for 4 hours. Then, cells were lysed with QIAzol Lysis Reagent (QIAGEN), and RNA was isolated using the miRNeasy Mini kit (QIAGEN). RNA quality was analyzed by the Agilent 2100 Electrophoresis Bioanalyzer system using the TapeStation Analysis software 4.1 (Agilent Technology Inc.), yielding RNA integrity number values of 10, 9.1, and 9.8 for the samples corresponding to mock-, FMDV- and FMDV-ΔL–infected cells, respectively. The small RNA libraries from 1 μg of RNA extracted of each sample were generated using the NEBNext Small RNA Library Prep Set for Illumina (Multiplex Compatible) kit (New England Biolabs) according to the manufacturer’s instructions. Then, the small RNA libraries were pooled in equal ratio, and the 175 bp fraction (98 to 288 bp) was extracted from a polyacrylamide gel (5 % mini-PROTEAN TBE precast gel, Bio-Rad). The quality of the resulting cDNA library was analyzed using High Sensitivity D1000 ScreenTape (Agilent Technology Inc.) and sequenced in a single lane of the NovaSeq 6000 (Illumina) using a NovaSeq 6000 S2 Reagent kit v1.5 (100 cycles). The RNA sequencing and construction of small RNA libraries were performed by the Genomics Unit at the Madrid Science Park Foundation [Universidad Autónoma de Madrid (UAM), Madrid, Spain].

The quality analysis of reads from the corresponding fastq data files was first performed using FastQC software. Trimmed and filtered reads between 18 and 28 nt in length were obtained from the raw data reads by removing the adaptor sequences and low-quality reads containing more than 20% bases with a quality score < 20 using the Trimmomatic tool. The small RNA read counting for each normalized length distribution (counts per million) was performed using the edgeR^1^ library in R software using a custom R script. Then, 18- to 28-nt and 22-nt small RNA read count from all samples were separately aligned to the FMDV O1K genome sequence (accession numbers D10138 and X00871, NCBI GenBank) using the Bowtie 2 aligner. The length and abundance distribution of 18- to 28-nt and 22-nt reads of positive- or negative-stranded vsRNAs from the libraries corresponding to cells infected with O1K or O1K-Lb viruses were analyzed using Bedtools through a custom script written in R, and the average coverage of each alignment was calculated and graphically plotted using the GenomeCoverageBed software.

Pairs of complementary 22-nt vsRNAs in each library with different base-pairing lengths (distance categories) were identified using Bedtools and plotted using R software. These distance categories were defined as 0 for perfect base-paired 22-nt vsRNAs with blunt ends, −2 for pairs with 2-nt overhang at the 3′-end of each strand, or 20 for pairs with 20-nt overhang at the 5′-end (*56*). The next-generation sequencing (NGS) data analysis was performed by the Genomic and next core facility (GENGS) at the Centro de Biología Molecular Severo Ochoa (CSIC-UAM). The RNA sequencing data files of the NGS project have been deposited in the European Nucleotide Archive (ENA) database of the European Molecular Biology Laboratory–European Bioinformatics Institute (EMBL-EBI) with accession number PRJEB64251.

### Statistical analysis

Data were analyzed using GraphPad Prism Software LCC (version 9.3.1), and *P* values were determined by one-way analysis of variance (ANOVA) test with Tukey’s post hoc correction for multiple comparisons. *P < 0.05, **P < 0.01, ***P < 0.001, and ****P < 0.0001.
