# Supplementary material for: Dicer is cleaved by the Leader protease encoded by foot-and-mouth disease virus to promote infection in mammalian cells
Source: Sci Adv. 2025 Jul 4;11(27):eadt3751. doi: 10.1126/sciadv.adt3751 (PMC12227051; doi:10.1126/sciadv.adt3751)
Supplement: Supplementary file 1 — Figs. S1 to S3 Table S1 [file sciadv.adt3751_sm.pdf]

Science Advances
AAAS

Supplementary Materials for

Dicer is cleaved by the Leader protease encoded by foot-and-mouth disease virus to promote infection in mammalian cells

Miguel Rodríguez-Pulido *et al.*

Corresponding author: Margarita Sáiz, msaiz@cbm.csic.es

Sci. Adv. **11**, eadt3751 (2025)
DOI: 10.1126/sciadv.adt3751

This PDF file includes:

Figs. S1 to S3
Table S1

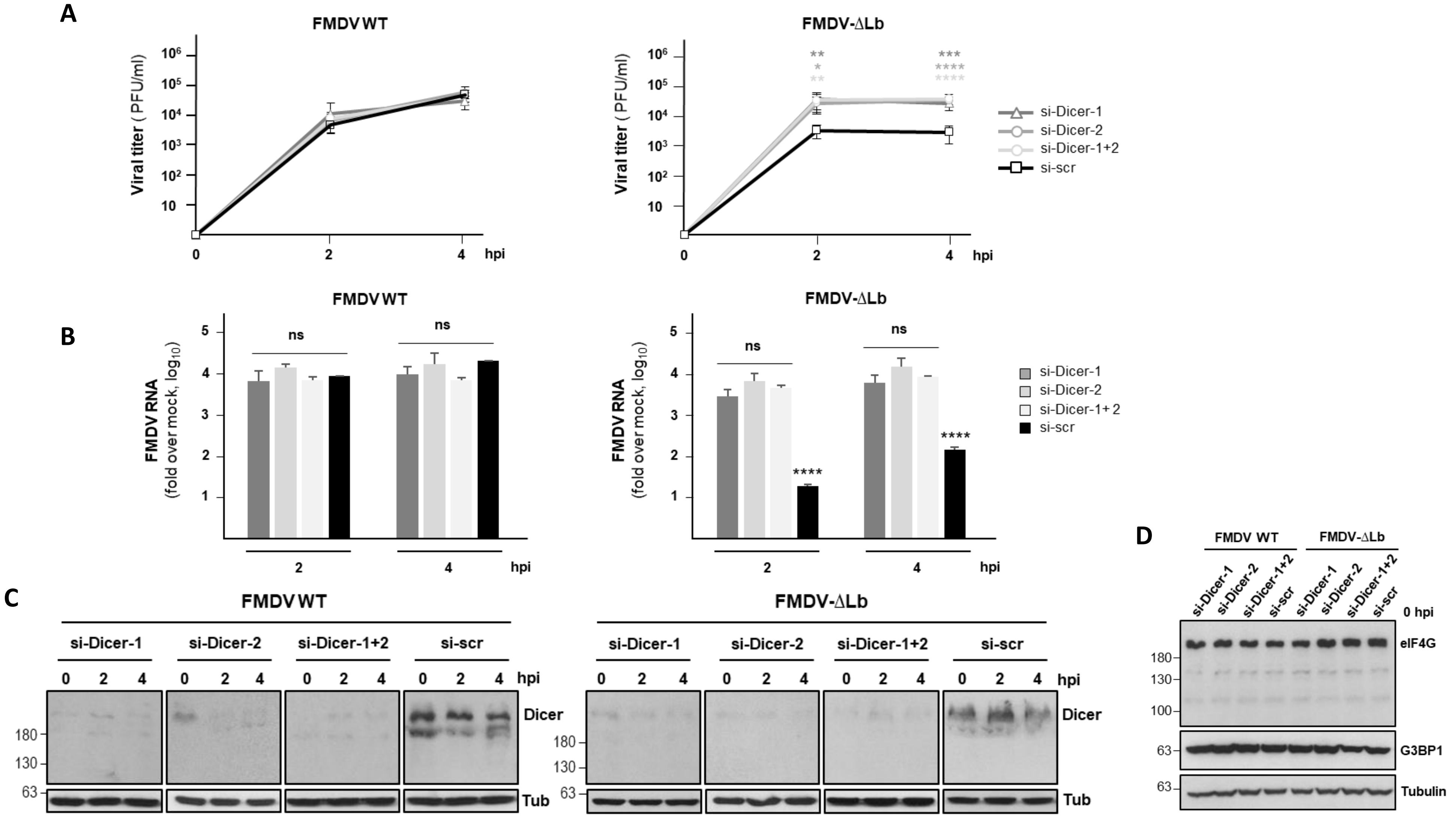

Fig. S1. Differential impact of Dicer silencing on FMDV WT and FMDV-ΔLb infection. Related to Fig. 3. IBRS-2 cells were transfected with two specific Dicer siRNAs independently (100 nM), a mixture of them (50 nM each) or a scramble siRNA (*55*) (100 nM) for 48 h. Then, cells were infected with FMDV WT or FMDV-ΔLb at an MOI of 5. At 2 or 4 h pi supernatants were collected for virus titration by plaque assay in BHK-21 cells (A) and cells lysed for quantification of viral RNA by RT-qPCR (*54*) (B) and WB (C to D). (C) Dicer expression levels at the indicated times pi. (D) Analysis of eIF4G and G3BP1 levels at 0 h pi. Data in A and B are mean ± SD of two independent experiments each performed in triplicate. ANOVA test with Dunnett's correction. *P < 0.05, **P < 0.01, ***P < 0.001 and ****P < 0.0001. Representative results are shown in C and D. siRNA Dicer-2 (5´- GGUAAGAGAACUACAGAAAUU-3´) targets nt 975-993 of swine Dicer (GenBank HQ184403.1).

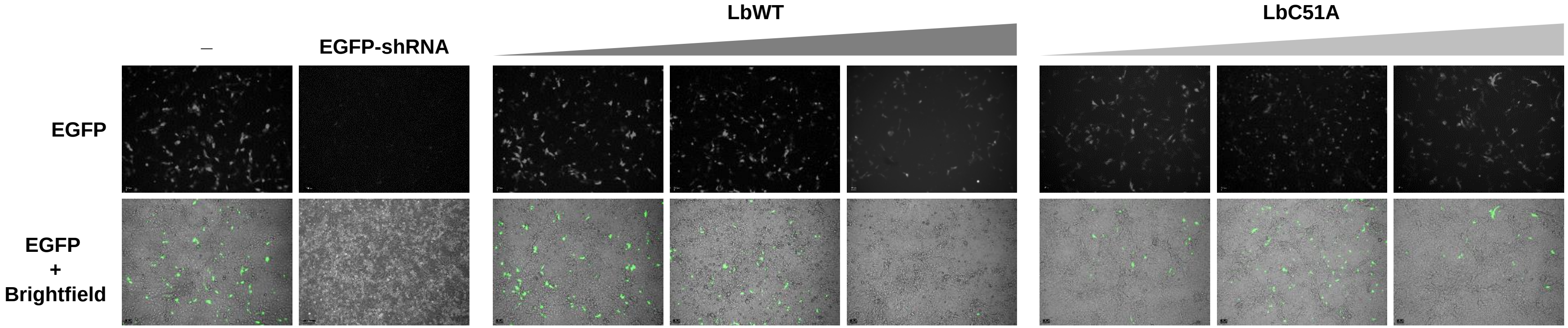

Fig. S2. Effect of Lpro on EGFP expression in IBRS-2 cells. Related to Fig. 4. IBRS-2 cells were transfected with a plasmid encoding EGFP (0.3 µg) alone or together with a specific EGFP shRNA (5 µg) or a plasmid encoding LbWT or C51A (0.2, 2 or 20 ng). At 48 h after transfection, cells were directly observed by fluorescence and bright-field microscopy and images were taken with a 10x magnification.

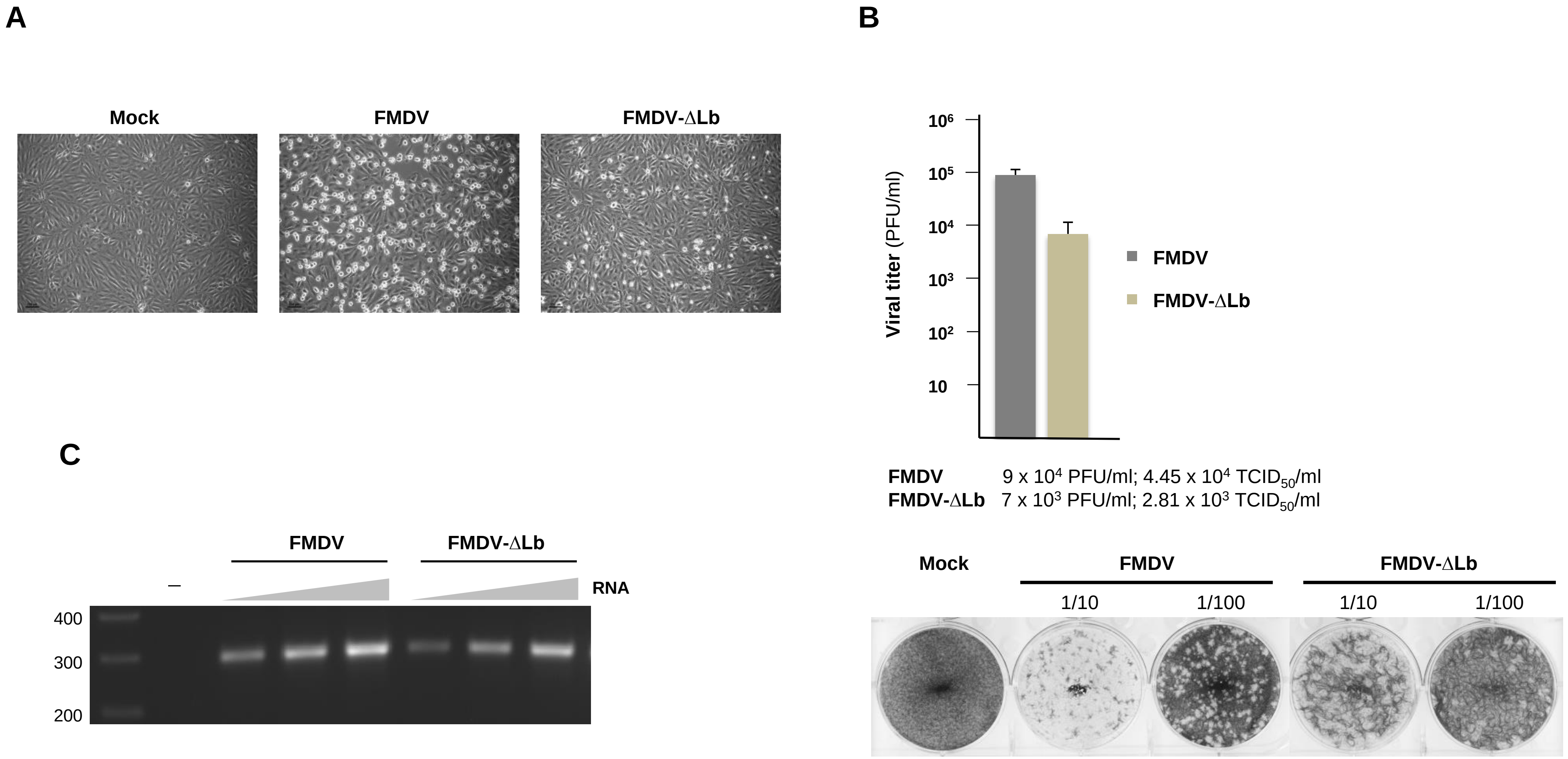

Fig. S3. Viral titers and cytopathic effect produced by viruses used in the RNAseq analysis. Related to Fig. 7. (A) IBRS-2 cells were mock-infected or infected with FMDV or FMDV-ΔLb at an MOI of 5 for 4 h. Images of the corresponding monolayers were captured at 4 h after infection. **(B)** Supernatants were collected at 4 h pi for viral titration in BHK-21 cells by plaque assay and 50% tissue culture infectious dose and expressed as PFU/ml and $TCID_{50}$/ml, respectively. The plaque phenotypes are shown for the 1/10 and 1/100 dilutions of supernatants in the lower panel. **(C)** IBRS-2 cells were collected at 4 h pi for RNA extraction and amplification of a 298-bp region of the FMDV RNA by RT-PCR using 10, 100 or 1000 ng of RNA as templates.

	FMDV	FMDV-ΔLb	Mock-infected
Total reads	52758820	46398789	41348679
Reads mapped to *Sus scrofa v11* genome[1]	19116254 (36.23%)	16203352 (34.92%)	14943018 (36.14%)
Unmapped reads	33275689 (63.07%)	30114647 (64.9%)	26405643 (63.86%)
Reads mapped to FMDV genome[2]	366877 (0.69%)	80790 (0.17%)	18 (0.00%)
Positive strand	364784 (99.43%)	77150 (95.49%)	14 (0.00%)
Negative strand	2093 (0.57%)	3640 (4.49%)	4 (0.00%)
FMDV reads of 18-28nt	82336 (22.44%)	23850 (29.52%)	0 (0.00%)
Positive strand	81404 (98.86%)	21934 (91.96%)	0 (0.00%)
Negative strand	932 (1.14%)	1916 (8.04%)	0 (0.00%)
FMDV reads of 21-23nt	15692 (4.27%)	5786 (7.16%)	0 (0.00%)
Positive strand	15427 (98.31%)	4957 (85.67%)	0 (0.00%)
Negative strand	265 (1.69%)	829 (14.33%)	0 (0.00%)
FMDV reads of 22nt	4552 (1.24%)	1812 (2.24%)	0 (0.00%)
Positive strand	4471 (98.22%)	1468 (81.01%)	0 (0.00%)
Negative strand	81 (1.78%)	344 (18.98%)	0 (0.00%)

Table S1. Profiles of viral small RNAs. Related to Fig. 7. The abundance of reads in each library for the different alignments, size range and polarity are shown as percentage in brackets. The ratio of positive and negative strand vsRNAs was calculated for the indicated size range. [1] GenBank assembly GCA_000003025.6; [2] GenBank D10138 and X00871.
